# Variabilidade da Pressão Arterial e do Risco Cardiovascular no ELSA-Brasil: Um Potencial Marcador Substituto para Predizer Mortalidade e Desfechos Cardiovasculares?

**DOI:** 10.36660/abc.20220525

**Published:** 2022-10-05

**Authors:** Maria Cristina Izar, Francisco A. H. Fonseca

**Affiliations:** 1 Universidade Federal de São Paulo Escola Paulista de Medicina São Paulo SP Brasil Universidade Federal de São Paulo Escola Paulista de Medicina , São Paulo , SP – Brasil

**Keywords:** Pressão Arterial, Homeostase, Doenças Cardiovasculares, Fatores de Risco, Hipertensão

A homeostase da pressão arterial (PA) é um elemento crucial na proteção de eventos cardiovasculares. Muitas diretrizes nacionais e internacionais ^1 - 4^ propuseram valores-alvo para a pressão arterial, e essas recomendações consideram metas ligeiramente diferentes de acordo com os estágios da hipertensão, estratificação de risco e presença de doenças renais ou cardiovasculares e lesão de órgão-alvo. No entanto, esses valores são baseados em medidas de consultório, monitorização ambulatorial da pressão arterial (MAPA) e monitorização domiciliar da pressão arterial (MDRA), não levando em conta a variabilidade da pressão arterial (VPA). A variabilidade interindividual da PA é um fator de risco independente para doenças cardiovasculares independentemente da PA média. ^5 - 7^ As flutuações nas medidas fisiológicas da pressão arterial não ocorrem aleatoriamente e podem contribuir ou ser preditores de desfechos cardiovasculares.

A maioria dos estudos avaliou a VPA em curto (24h), médio (>2 dias) ou longo prazo (semanal, mensal ou anual). ^8 , 9^ A VPA de curto prazo pode estar associada ao aumento do risco cardiovascular. ^10 , 11^

No estudo de Zarife et al., ^12^ os autores utilizaram dados basais de 14.357 participantes do ELSA-Brasil sem história prévia de doença cardiovascular.

A VPA foi quantificada em uma única visita na linha de base pelo coeficiente de variação de três medidas padronizadas de pressão arterial sistólica usando um oscilômetro validado (Omron HEM 705CPINT) e correlacionado com o risco de DCVA. A VPA foi dividida em quartis, e o quartil mais alto foi associado a um risco cardiovascular significativamente maior em homens e mulheres. O sexo masculino apresentou maior risco cardiovascular do que o feminino em todos os quartis, sendo a maior diferença observada no quarto quartil do VPA. Além disso, a comparação dos quartis por sexo mostrou risco significativamente maior para os homens no terceiro e quarto quartis e no quarto quartil para as mulheres. A VPA também foi associada a maior velocidade de onda de pulso, menor taxa de filtração glomerular e hipercolesterolemia. Nenhum estudo relatou avaliação de risco cardiovascular e VPA em uma única visita. Os resultados do ELSA-Brasil, nesta grande coorte prospectiva, sugerem que este pode ser um marcador de risco de doença cardiovascular e ajudar a identificar pacientes que necessitam de acompanhamento mais próximo ou terapia mais intensiva. Vale ressaltar que a maioria dos participantes do ELSA-Brasil era de indivíduos normotensos (64%), reforçando o conceito de que a pressão arterial é uma medida contínua de risco e que a VPA pode ser importante não apenas para os hipertensos, mas também pode ser avaliada em indivíduos com medidas normais de pressão arterial. Outros passos devem ser a avaliação da VPB em visita única e dos desfechos cardiovasculares no ELSA-Brasil. no ELSA-Brasil.
