# Supplementary material for: Variabilidade da Pressão Arterial e do Risco Cardiovascular no ELSA-Brasil: Um Potencial Marcador Substituto para Predizer Mortalidade e Desfechos Cardiovasculares?
Source: Arq Bras Cardiol. 2022 Oct 5;119(4):512–3. [Article in Portuguese] doi: 10.36660/abc.20220525 (PMC9563885; doi:10.36660/abc.20220525)
Supplement: Translation

# Translation: Blood Pressure Variability and Cardiovascular Risk in ELSA-Brasil: A Potential Surrogate Marker for Predicting Mortality and Cardiovascular Outcomes?

**Authors:** Maria Cristina Izar, Francisco A. H. Fonseca

**Affiliations:** 1 Brazil Universidade Federal de São Paulo Escola Paulista de Medicina, São Paulo, SP – Brazil

**Keywords:** Blood Pressure, Homeostasis, Cardiovascular Diseases, Risk Factors, Hypertension

Blood pressure (BP) homeostasis is a crucial element in the protection of cardiovascular events. Many national and international guidelines^1 - 4^ have proposed target values for blood pressure, and these recommendations consider slightly different goals according to hypertension stages, risk stratification and presence of renal or cardiovascular diseases, and target organ lesion. However, these values are based on office measures, ambulatory blood pressure monitoring (ABPM) and home blood pressure monitoring (HBPM), not accounting for blood pressure variability (BPV). BP intraindividual variability is an independent risk factor for cardiovascular diseases regardless of mean BP.^5 - 7^ Fluctuations in physiological measures of blood pressure do not occur randomly and can contribute or be predictors of cardiovascular outcomes.

Most studies evaluated BPV on short (24h), medium (>2 days) or long-term (weekly, monthly, or annually).^8 , 9^ Short-term BPV can be associated with increased cardiovascular risk.^10 , 11^

In the study by Zarife et al.,^12^ the authors used baseline data from 14,357 participants of ELSA-Brasil without prior history of cardiovascular disease.

BPV was quantified in a single visit at baseline by the coefficient of variation of three standardized systolic blood pressure measurements using a validated oscilometer (Omron HEM 705CPINT) and correlated with ASCVD risk. BPV was divided into quartiles, and the highest quartile was associated with a significantly higher cardiovascular risk in both men and women. Males had a higher cardiovascular risk than females in all quartiles, with the greatest difference observed in the fourth BPV quartile. In addition, comparing quartiles by sex showed a significantly higher risk for men in the third and fourth quartiles and the fourth quartile for women. BPV was also associated with higher pulse-wave velocity, lower glomerular filtration rate, and hypercholesterolemia. No studies have reported cardiovascular risk assessment and BPV in a single visit. The results from ELSA-Brasil, in this large prospective cohort suggest that this can be a marker of cardiovascular disease risk and help identify patients needing closer monitoring or more intensive therapy. It is worthy to note that the majority of participants of ELSA-Brasil were normotensive individuals (64%), reinforcing the concept that blood pressure is a continuous measure of risk and that BPV can be important not only for those with hypertension but can be assessed in subjects with normal blood pressure measures. Further steps should be assessing single visit BPV and cardiovascular outcomes in ELSA-Brasil.
